# Two New Flavones from *Tridax procumbens* Linn

**DOI:** 10.3390/molecules15096357

**Published:** 2010-09-09

**Authors:** Runsheng Xu, Jing Zhang, Ke Yuan

**Affiliations:** 1 Nurturing Station for the State Key Laboratory of Subtropical Silviculture, Zhejiang Agriculture and Forestry University, Linan 311300, China; 2 College of Chemistry and Life Science, Zhejiang Normal University, Jinhua 321004, China; 3 College of Pharmacy, Henan University of Traditional Chinese Medicine, Zhengzhou 450008, China

**Keywords:** *Tridax procumbens* Linn, 8,3′-dihydroxy-3,7,4′-trimethoxy-6-*O*-*β*-D- glucopyranosyl flavone, 6,8,3′-trihydroxy-3,7,4′-trimethoxyflavone, antioxidant activity, DPPH, FRAP

## Abstract

Two new flavones, 8,3′-dihydroxy-3,7,4′-trimethoxy-6-*O*-*β*-D-glucopyranosyl flavone (**1**) and 6,8,3′-trihydroxy-3,7,4′-trimethoxyflavone (**2**) were isolated from *Tridax procumbens* Linn*.*, together with the four known compounds puerarin (**3**), esculetin (**4**), oleanolic acid (**5**) and betulinic acid (**6**). The structures of the two new flavones were elucidated based on chemical analysis and spectral methods (IR, 1D and 2D NMR, ESI-MS, HR-ESI-MS). The antioxidant activity of the two new flavones were evaluated by two methods, the 1,1-diphenyl-2-picrylhydrazyl (DPPH) radical scavenging activity and ferric reducing antioxidant power (FRAP) assays, and the data showed that compounds **1** and **2** have certain antioxidant activity, with the antioxidant activity of compound **2** being stronger than that of compound **1**.

## 1. Introduction

**Figure 1:**
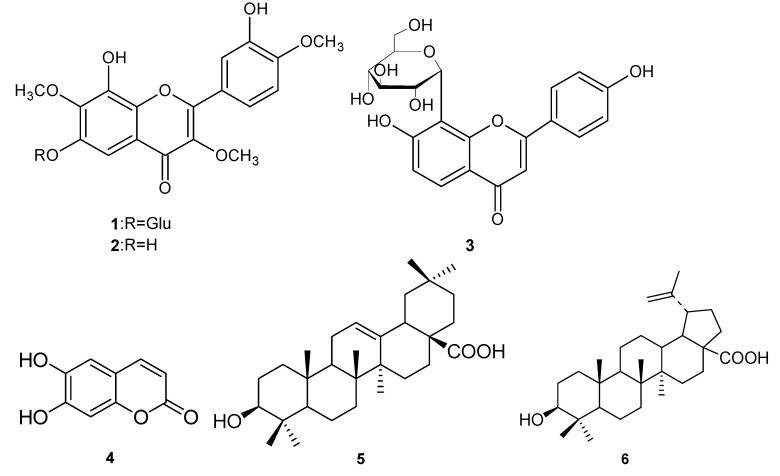
Structures of compounds **1-6**.

*Tridax procumbens* Linn., growing in the tropical areas of the Americas, belongs to the Compositae family [1]. It was introduced into China in the 1940s [2], and has been used in traditional medicine by the native populations to treat **bronchitis**, dysentery, diarrhoea and to prevent hair loss. Recently, the chemical components of its essential oils have been investigated [3,4]. In this article, we report the isolation and structural identification of six compounds, including three flavones: 8,3′-dihydroxy-3,7,4′-trimethoxy-6-*O*-*β*-D-glucopyranosyl flavone (**1**), 6,8,3′-trihydroxy-3,7,4′-trimethoxyflavone (**2**), and puerarin (**3**) [5], one coumarin, esculetin (**4**) [6], and two triterpenoids: oleanolic acid (**5**)** [7]** and betulinic acid (**6**) [8] (Figure 1). Among them, compounds **1** and **2** are two new flavones and compounds **3~6** were isolated from this species for the first time. This paper presents the isolation and structural elucidation of compounds **1** and **2,** and their antioxidant activity, evaluated using the well known DPPH and FRAP assays.

## 2. Results and Discussion

**Figure 2:**
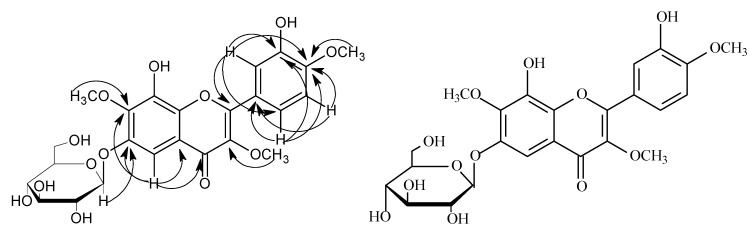
The key HMBC correlations of compound **1**.

Compound **1** was obtained as a yellow amorphous powder. The HR-ESI-MS showed an ion at *m/z* 545.1274 [M+Na]^+^ (calcd 545.1271), corresponding to the molecular formula, C_24_H_26_O_13_. The ^1^H- NMR spectrum displayed three aromatic protons at *δ* 7.65 (1H, dd, *J* = 8.0, 2.0 Hz), 7.03 (1H, d, *J* = 8.0 Hz) and 7.60 (1H, d, *J* = 2.0 Hz), suggesting an ABX spin system in the B-ring [9], a signal of an isolated aromatic proton at *δ* 6.87 (s, 1H) was also seen. Three methoxy groups appeared at *δ* 3.80 (1H, s), 3.88 (s, 1H), 3.93 (1H, s). The sugar protons appeared between *δ* 3.41~5.10. The doublet peak of one single proton at *δ* 5.10 (1H, d, *J* = 7.4 Hz) was attributed to the H-1′′, and suggested that the D-glucose moiety was in a *β*-orientation. The ^13^C-NMR spectrum (Table 1) indicated that compound **1** was a flavonol glycoside with twenty-four carbons, including fifteen flavonoid carbons and six sugar carbons, with *δ* 102.0 being a characteristic signal for the C-1′′of the sugar. Acid hydrolysis of compound **1** gave glucose, identified by paper chromatography and comparison with an authentic sample. A HSQC experiment showed a correlation between the proton signal at H-1′′ (*δ* 5.10) and the carbon signal at C-1′′ (*δ* 102.0). The location of the glucose was established on the basis of HMBC correlations from H-1′′ (*δ* 5.10) to C-6 (*δ* 157.9) (Figure 2). The *β*-D-Glycosyl was attached to the C-6 of A-ring. The ^1^H-NMR and ^13^C-NMR data of the D-Glycosyl was in agreement with those of a reference. The HMBC spectrum showed the three methoxy groups were linked to C-3, C-7 and C-4′ of the flavonoid skeleton. On the basis of the above evidence, The chemical structure of compound **1** was formulated as: 8,3′-dihydroxy-3,7,4′-trimethoxy-6-*O*-*β*-D-glucopyranosyl flavone.

**Table 1:** ^1^H-NMR and ^13^C-NMR data for compounds **1** and **2 in CD_3_OD**, *δ* (ppm), *J* (Hz).

Position	Compound 1			Compound 2		
	***δ* H**	***δ* C**	**HMBC**	***δ* H**	***δ* C**	**HMBC**
2		158.3			115.9	
3		139.9			138.8	
4		180.4			179.4	
5	6.87 s	95.6	C10, C7, C9, C6, C4	6.54 s	93.2	C10, C7, C9
6		157.9			155.1	
7		133.8			130.1	
8		153.8			152.4	
9		153.4			151.9	
10		108.1			106.4	
1′		124.1			123.7	
2′	7.60 d (2.0)	116.3	C6′, C3′, C4′, C2	7.73 d (2.0)	114.6	C6′, C3′, C4′, C2
3′		147.7			145.6	
4′		151.9			148.9	
5′	7.03 d (8.0)	112.3	C1′, C3′, C4′	6.97 d (8.0)	110.5	C1′, C3′, C4′
6′	7.65 dd (8.0, 2.0)	122.4	C2′, C4′	7.71 dd (8.0, 2.0)	121.7	C2′, C4′
3-OCH_3_	3.80 s	60.6	C3	3.86 s	60.3	C3
7-OCH_3_	3.88 s	61.5	C7	4.04 s	61.1	C7
4′-OCH_3_	3.93 s	56.4	C4′	3.99 s	56.2	C4′
1′′	5.10 d (7.4)	102.0	C6			
2′′	3.49 m	74.8	C1′′, C3′′			
3′′	3.53 dd	78.0	C2′′, C4′′			
4′′	3.41 m	71.3	C3′′, C5′′			
5′′	3.42 m	78.5	C4′′			
6′′	3.69 dd, 3.95 m	62.6	C5′′			

**Figure 3:**
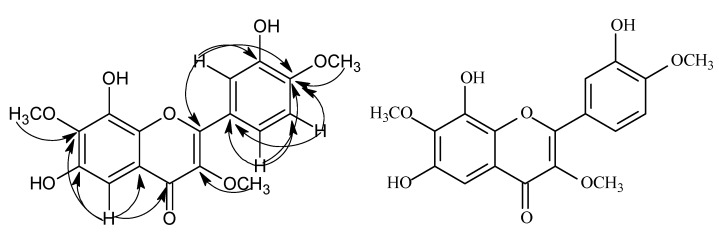
The key HMBC correlations of compound **2**.

Compound **2** was obtained as a yellow amorphous powder. The HR-ESI-MS showed an [M-H]^-^ ion at *m/z* 359.0709 (calcd 359.0748), corresponding to the molecular formula, C_18_H_16_O_8_. The ^1^H-NMR spectrum showed three aromatic protons at *δ* 7.71 (1H, dd, *J* = 8.0, 2.0 Hz), 6.97 (1H, d, *J* = 8.0 Hz) and 7.73 (1H, d, *J* = 2.0 Hz) suggesting an ABX spin system in the B-ring, and an isolated aromatic proton signal at *δ* 6.87 (s, 1H) was also seen. Three methoxy groups were noted at *δ* 3.80 (s, 3H), 3.88 (3H, s), 3.93 (3H, s). The ^1^H-^1^H COSY spectra of compound **2** disclosed that the signal at *δ* 6.97 (1H, d, *J* = 8.0 Hz) was correlated to the one at *δ* 7.71 (1H, d. *J* = 8.0, 2.0 Hz). In the HMBC, H-1′′ signal at *δ* 5.10 correlated to C-6 (*δ* 157.9, Figure 3). The HMBC spectrum showed the three methoxy groups were linked to C-3, C-7 and C-4′ of the flavonoid skeleton. On the basis of the above evidence, the chemical structure of compound **2** was formulated as: 6,8,3′-trihydroxy-3,7,4′-trimethoxyflavone.

### Antioxidant activity

**Figure 4:**
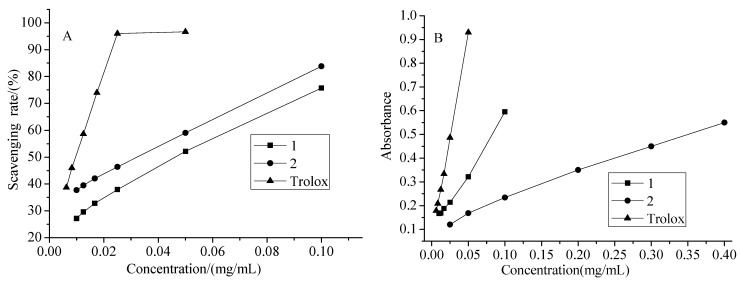
The antioxidant activity of compounds **1**, **2** and Trolox with different concentrations (**A:** DPPH assay, **B:** FRAP assay).

The antioxidant activities of compounds **1** and **2** were evaluated using the DPPH and FRAP assays. The DPPH assay has been widely used to test the free radical scavenging ability of plant components. In the present study, it was found that both the two new compounds had DPPH free-radical scavenging activity. The DPPH scavenging rates are shown in Figure 4(A), and the IC_50_ values are shown in Table 2. From these data, it can be concluded that the sequence of antioxidant activity of these compounds was as follows: Trolox (reference) > compound **1** > compound **2**. The FRAP assay can reflect the antioxidant activity through the variation of absorbance at a certain wavelength. The absorbances of the two new compounds are shown in Figure 4(B), and TEAC values are shown in Table 2. These TEAC values demonstrated that the sequence of reducing power of these compounds was as follows: Trolox > compound **1** > compound **2**.

**Table 2:** IC_50_ values and TEAC values of compounds **1**, **2** and Trolox with different concentration.

	Compound 1	Compound 2	Trolox
**IC_50_ value (mg/mL)**	0.03180	0.04618	0.00948
**TEAC value (mg/g)**	0.29784	0.07017	1.00000

## 3. Experimental

### 3.1. General

1D and 2D NMR spectra (chemical shifts in ppm, coupling constants in Hz) were recorded on a Bruker DPX 400 instrument. The ^1^H NMR spectra were reported in delta (*δ*) units, parts per million (ppm) downfield from the internal standard. Coupling constants are reported in Hertz (Hz). ESI mass spectra were obtained on a Waters Q-Tof Micro TM mass spectrometer coupled to a Waters 2795 system that included an online photodiode array detector. HR-ESI-MS spectra were determined on a Waters Q-Tof MicroTM ESI-MS. The compounds were detected from their UV absorbance and by spraying on TLC plates with FeCl_3_-K_3_[Fe(SCN)_6_] reagent.

### 3.2. Plant aaterial

The whole plants of *Tridax procumbens* was collected in Sanya city, Hainan Province, China, in September 2007. The plant was identified by Prof. Shi-man Huang of Hainan University. A voucher specimen of this plant (ZJFU-20070910-10) has been deposited in our laboratory.

### 3.3. Extraction and isolation [10]

Air-dried and powdered whole plants of *Tridax procumbens* (5 kg) was extracted with 70% EtOH (20 L × 3) at room temperature for 24 h. The combined EtOH extracts was concentrated by vacuum thin film evaporation below 50 °C. After concentration the extracts were dissolved in H_2_O (5 L) and extracted successively with petroleum ether (15 L × 3) and EtOAc (15 L × 3). The EtOAc layer was separated by repeated column chromatography on Sephadex LH-20, RP-18 using MeOH/H_2_O as eluant to produce six fractions. Fr. 4 (15.5 g) was eluted with MeOH-H_2_O (40:60) and was then repeatedly subjected to column chromatography (CC) to yield 8,3′-dihydroxy-3,7,4′-trimethoxy-6-*O*-*β*- D-glucopyranosyl flavone (**1**, 85.6 mg) and 6,8,3′-trihydroxy-3,7,4′-trimethoxyflavone (**2**, 51.0 mg). Puerarin (**3**, 68.2 mg), esculetin (**4**, 27.4 mg), oleanolic acid (**5**, 58.5 mg) and betulinic acid (**6**, 35.5 mg).

### 3.4. Spectral data

Compound **1**: 35.6 mg, yellow amorphous powder, mp: 206–208 °C (MeOH); [α]_D_^20^ = -45.6(c 0.30, MeOH); UV *λ*_max_ nm (log*ε*): 231 (3.80), 291 (3.65); IR *υ*_max_ cm^-1^ (KBr): 3432 (OH), 1600, 1545, 1456, 1260, 1223, 829 cm^-1^; HR-EI-MS *m/z* 545.1274 [M+Na]^+^ (Calcd 545.1254); ^1^H-NMR, ^13^C-NMR and 2D experiments see Table 1.

Compound **2**: 51.0 mg, yellow amorphous powder, mp: 195–197 °C (MeOH); [α]_D_^20^ = -49.5(c 0.30, MeOH); UV *λ*_max_ nm (log*ε*): 254 (3.82), 275 (3.66), 277 (1.98); IR υ_max_ cm^-1^ (KBr): 3400 (OH), 1655, 1600, 1545, 1456, 829 cm^-1^; HR-EI-MS *m/z* 359.0709 (Calcd 359.0748); ^1^H-NMR, ^13^C-NMR and 2D experiments see Table 1.

### 3.5. Process of acid hydrolysis

The process of acid hydrolysis was: compound **1** (30 mg) was dissolved in 2% sulfuric acid aqueous solution (30 mL) in a 100 mL besker by heating of electric furnace. Solution was heated for one hour in boiling condition and then filtered. the filter residue was aglycone. The concentrated hydrolysate was identified by paper chromatography by D-glucose as authentic sample and B:A:W (4:1:5) as the solvent system. The sugars was identified as D-glucose. Aglycone was yellow powder, mp 235–236 °C.

### 3.6. DPPH and FRAP assays

#### 3.6.1. DPPH assay

The DPPH radical scavenging activity was evaluated according to the literature [11] with the slight modification. Briefly, each sample (100 µL of various concentrations) was added to ethanolic DPPH solution (100 µL, 2.5 × 10^-2^ mg/mL). After mixing gently and standing at 24 °C for 30 min, the absorbances were measured at 530 nm using a VERSAmax microplate reader spectrophotometer. The percentage of DPPH which was scavenged was calculated using the following formula:

Scavenging %= 1- (Ap-Ac) /Amax × 100%.

where A_p_ was the stable absorbance of DPPH ethanol solution (100 μL) plus sample solution (100 μL), A_c_ was the stable absorbance of 70% ethanol solution (100 μL) plus sample solution (100 μL) and A_max_ was the stable absorbance of DPPH ethanol solution (100 μL) plus 70% ethanol solution (100 μL).

#### 3.6.2. FRAP assay [12]

FRAP assay was carried out in a microtiter plate. Each sample (80 μL) with appropriate dilution if necessary was added to FRAP reagent [13] (150 μL, 10 parts of 0.3 mol/L sodium acetate buffer at pH 3.7, 1 part of 0.01 mol/L TPTZ solution, and 1 part of 0.02 mol/L FeCl_3_^.^ 6H_2_O solution), and 70% ethanol (80 μL) plus FRAP reagent (150 μL) was used as a blank, then the wavelength at 593 nm was read after 10 min. Fresh working solutions of Trolox were used for calibration, and the total antioxidant capacity were expressed as TEAC (Trolox Equivalent Antioxidant Capacity) values, it was calculated when the absorbency at 0.4 TEAC value means mg TE/mg flavone.

## 4. Conclusions

In our present study, two new flavones **1-2**, together with four known compounds **3~6** were separated from the aerial parts of *Tridax procumbens*, and their structures were identified by chemical analysis and extensive spectral evidence, including IR, 1D and 2D NMR, ESI-MS, HR-ESI-MS data. The antioxidant activity of the two new flavones were evaluated by the DPPH and FRAP assays, and the data showed that compounds **1** and **2** have certain antioxidant activity. The antioxidant activity of compound **2** was weaker than that of Trolox, the positive control, but stronger than that of compound **1**.

## Supplementary Materials

Supplementary materials can be accessed at http://www.mdpi.com/1420-3049/15/9/6357/s1.
