# Supplementary material for: Two New Flavones from Tridax procumbens Linn
Source: Molecules. 2010 Sep 9;15(9):6357–64. doi: 10.3390/molecules15096357 (PMC6257746; doi:10.3390/molecules15096357)

Supporting Information

Two New flavones from *Tridax procumbens* Linn.

Runsheng Xu [1, 2], Jing Zhang[3], Ke Yuan[1]*

[1] Research and Development Center of Natural Medicine, Zhejiang Agriculture and Forestry University, Linan 311300, China

[2] College of Chemistry and Life Science, Zhejiang Normal University, Jinhua 321004, China

[3] College of Pharmacy, Henan University of Traditional Chinese Medicine, Zhengzhou, 450008, China

S1. ^{1}H spectrum of **Compound 1** in CD3OD

S2. ^{13}C spectrum of **Compound 1** in CD3OD

S3. DEPT 135 spectrum of **Compound 1** in CD3OD

S4. ^{1}H-^{1}H COSY spectrum of **Compound 1** in CD3OD

S5. HSQC spectrum of **Compound 1** in CD3OD

S6. HMBC spectrum of **Compound 1** in CD3OD

S7. HR-ESI-MS spectrum of **Compound 1**

S8. ^{1}H spectrum of **Compound 2** in CD3OD

S9. ^{13}C spectrum of **Compound 2** in CD3OD

S10. DEPT 135 spectrum of **Compound 2** in CD3OD

S11. ^{1}H-^{1}H COSY spectrum of **Compound 2** in CD3OD

S12. HSQC spectrum of **Compound 2** in CD3OD

S13. HR-ESI-MS spectrum of **Compound 2**

S1. ^{1}H spectrum of **Compound 1** in CD_3OD

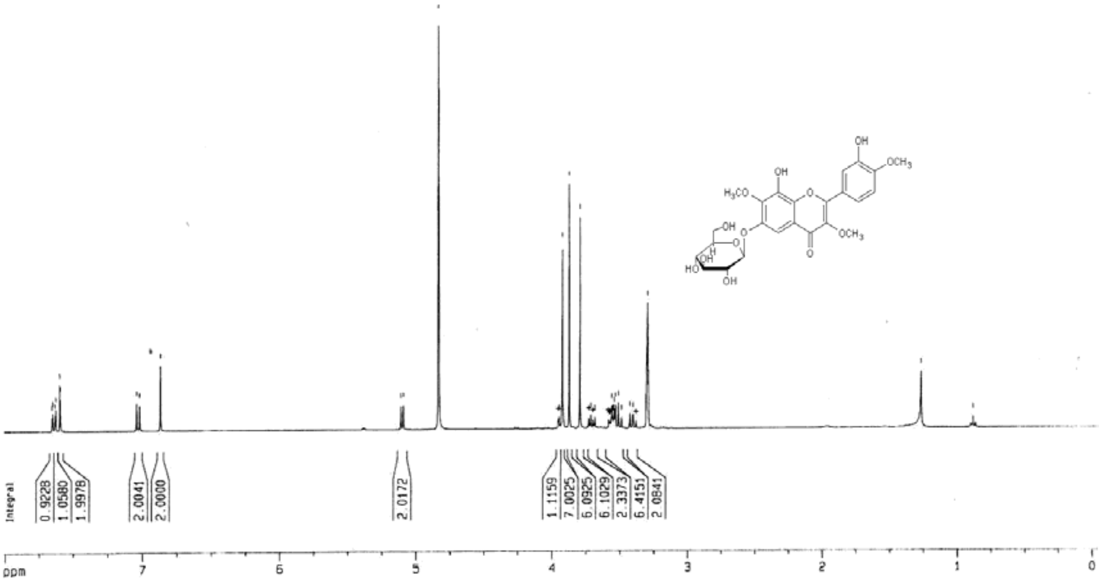

S2. ^{13}C spectrum of **Compound 1** in CD_3OD

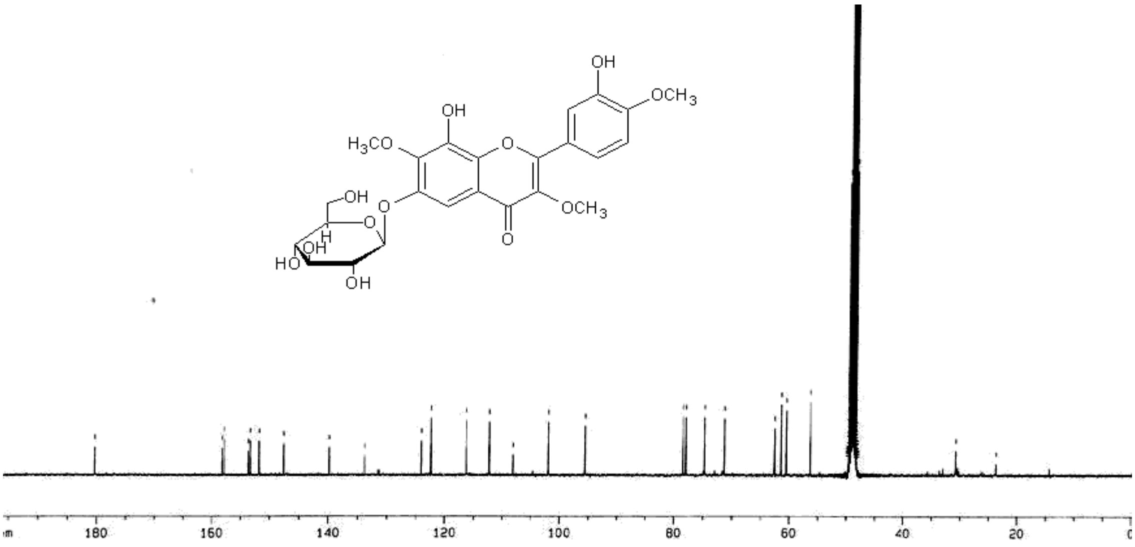

S3. DEPT 135 spectrum of **Compound 1** in CD_3OD

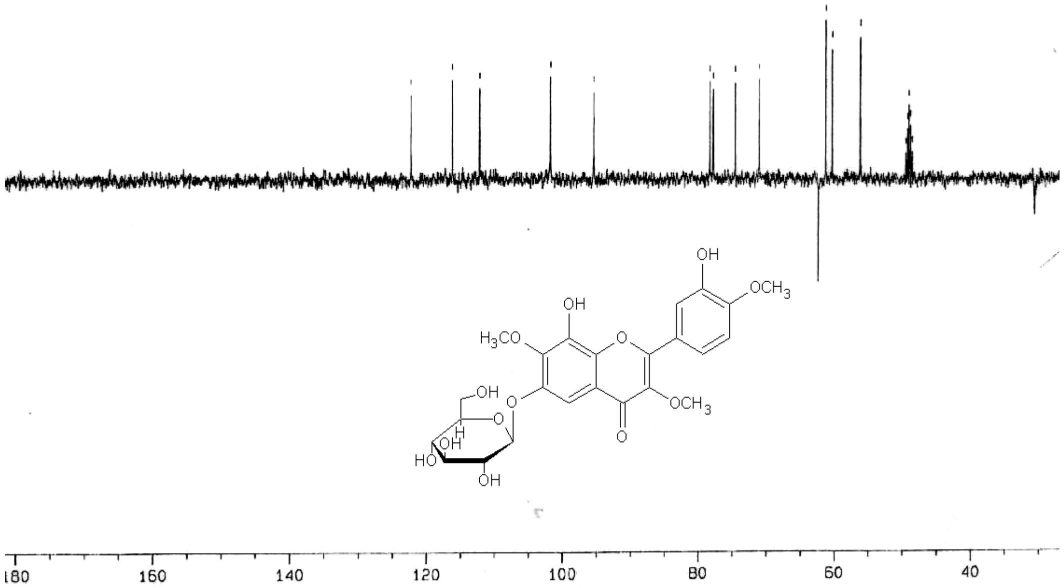

S4.^{1}H-^{1}H COSY spectrum of **Compound 1** in CD_3OD

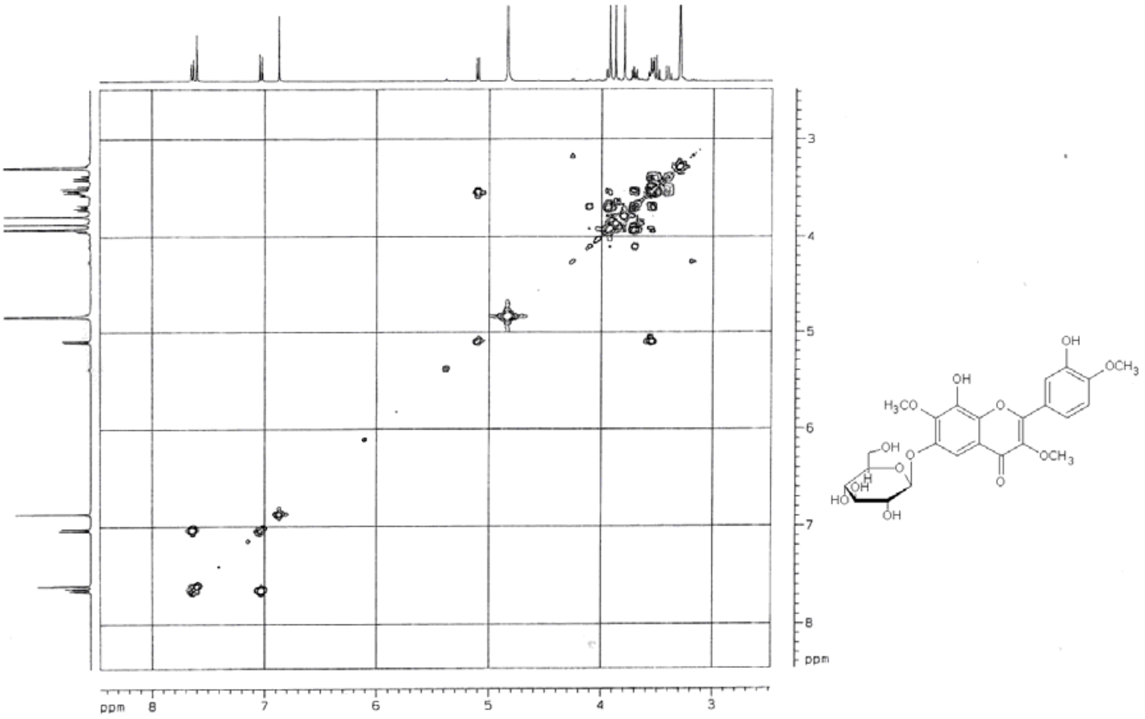

S5.HSQC spectrum of **Compound 1** in CD3OD

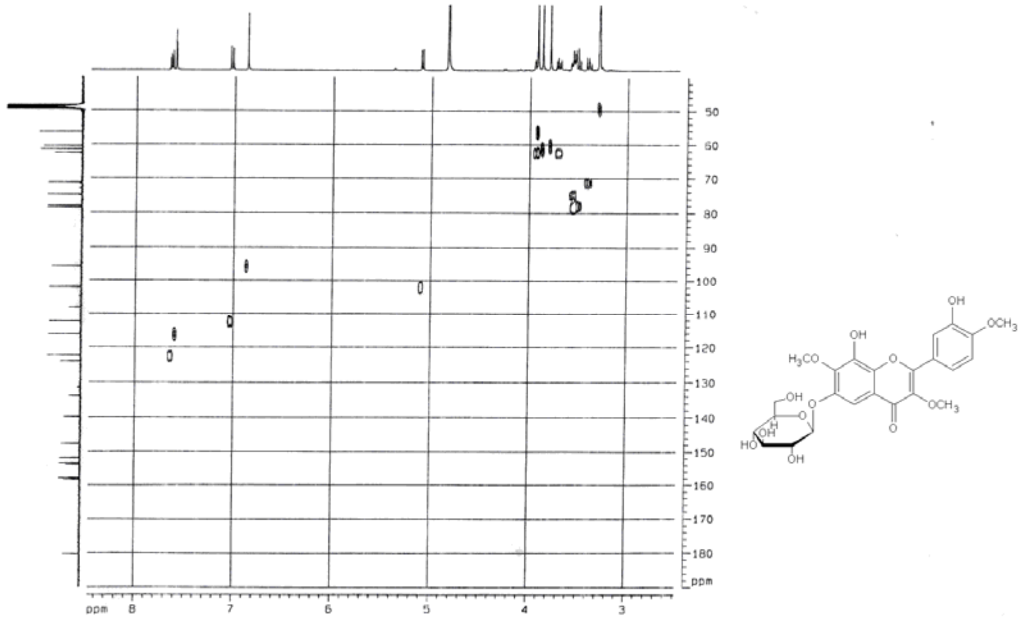

S6.HMBC spectrum of **Compound 1** in CD3OD

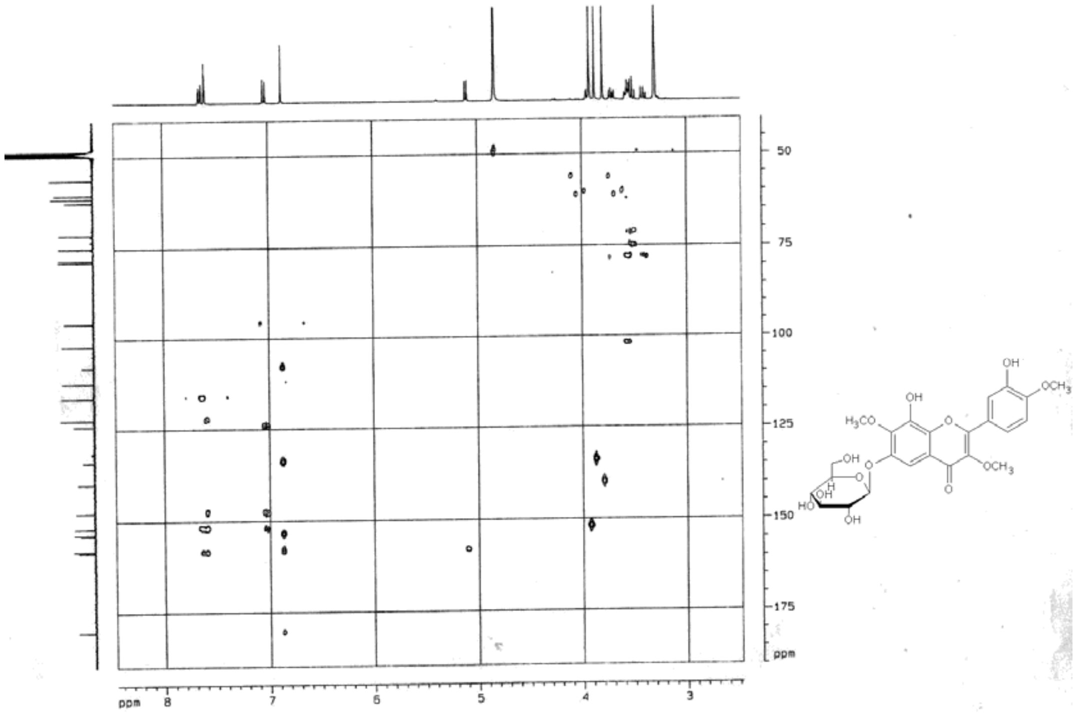

S7.HR-ESI-MS spectrum of **Compound 1**

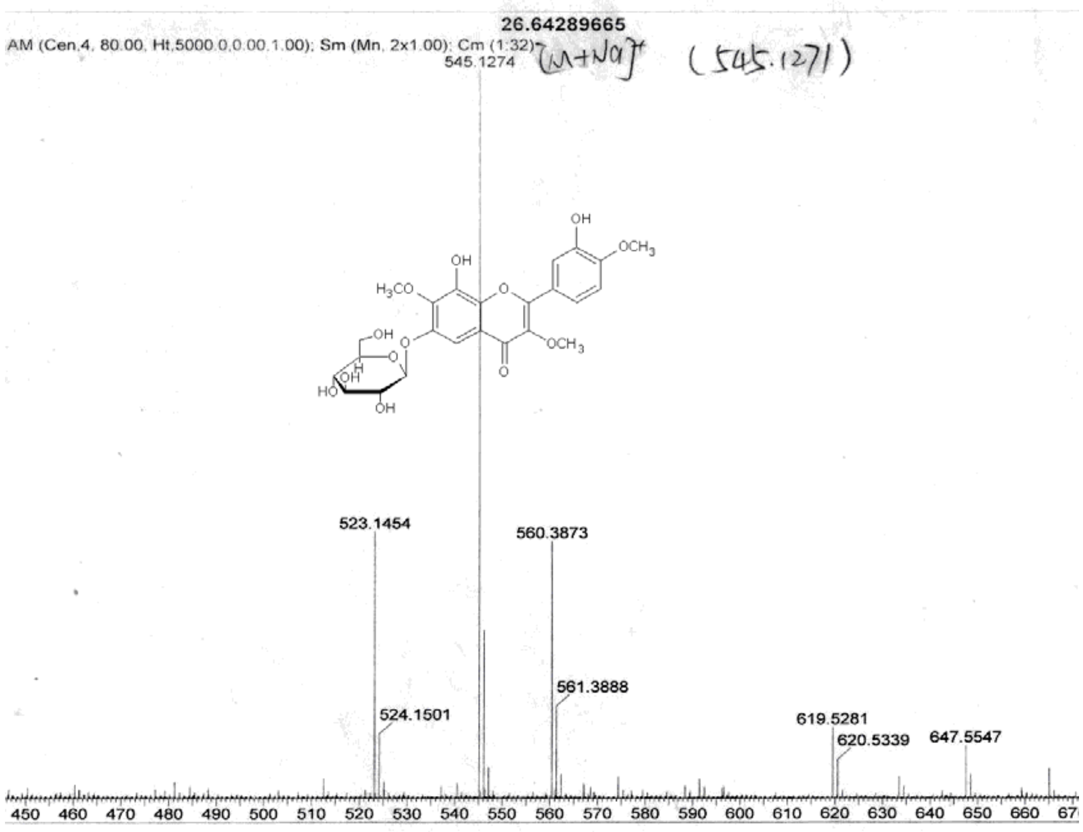

S8.^{1}H spectrum of **Compound 2** in CD3OD

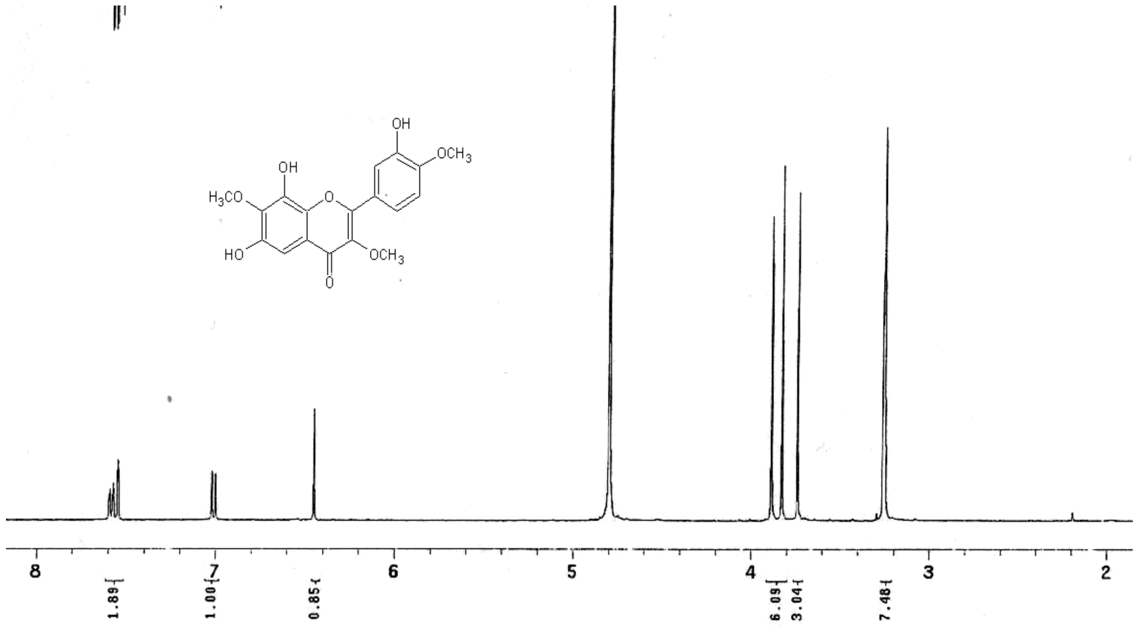

S9.^{13}C spectrum of **Compound 2** in CD_3OD

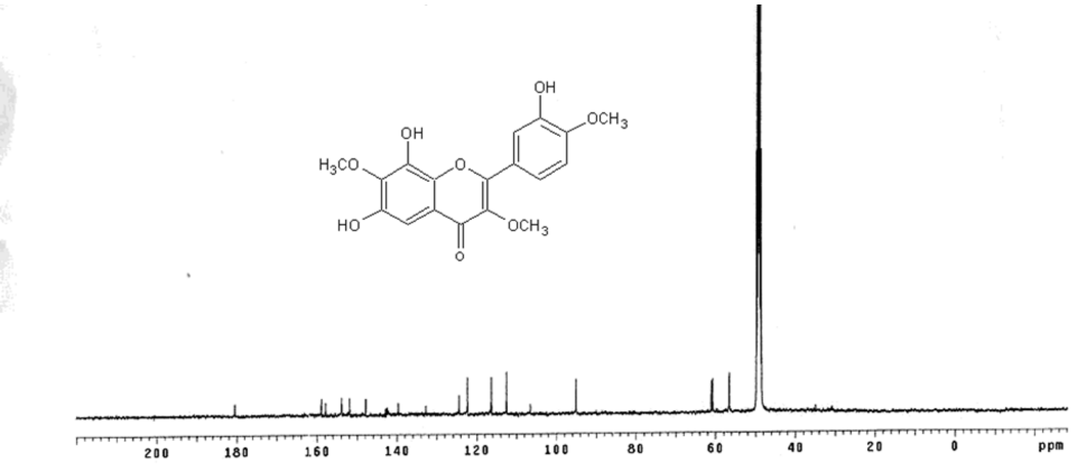

S10.DEPT 135 spectrum of **Compound 2** in CD_3OD

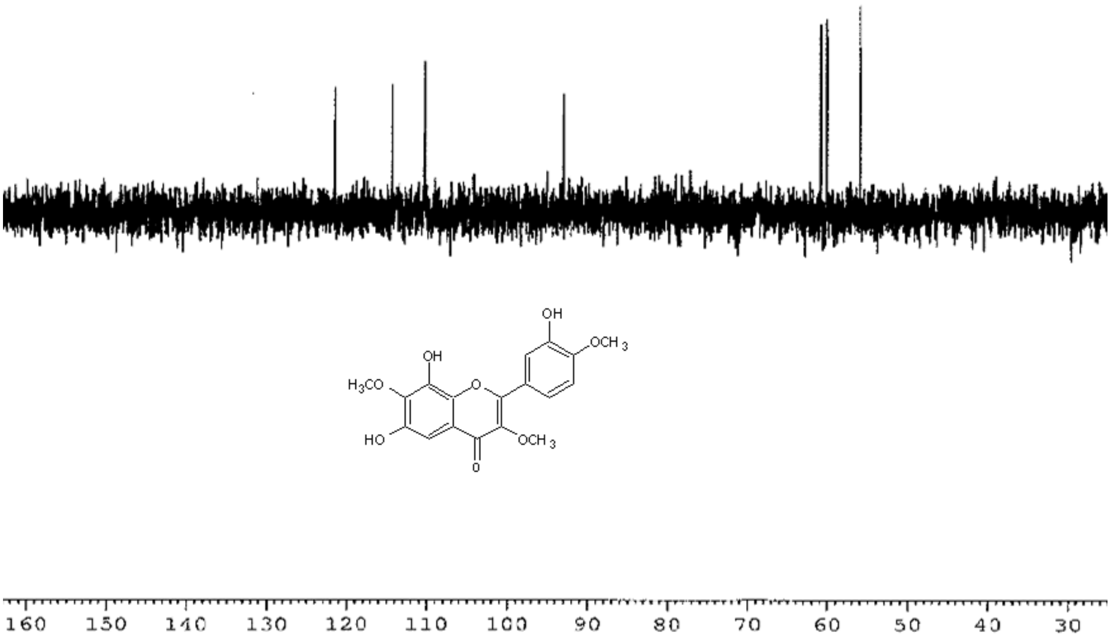

S11.^{1}H-^{1}H COSY spectrum of **Compound 2** in CD3OD

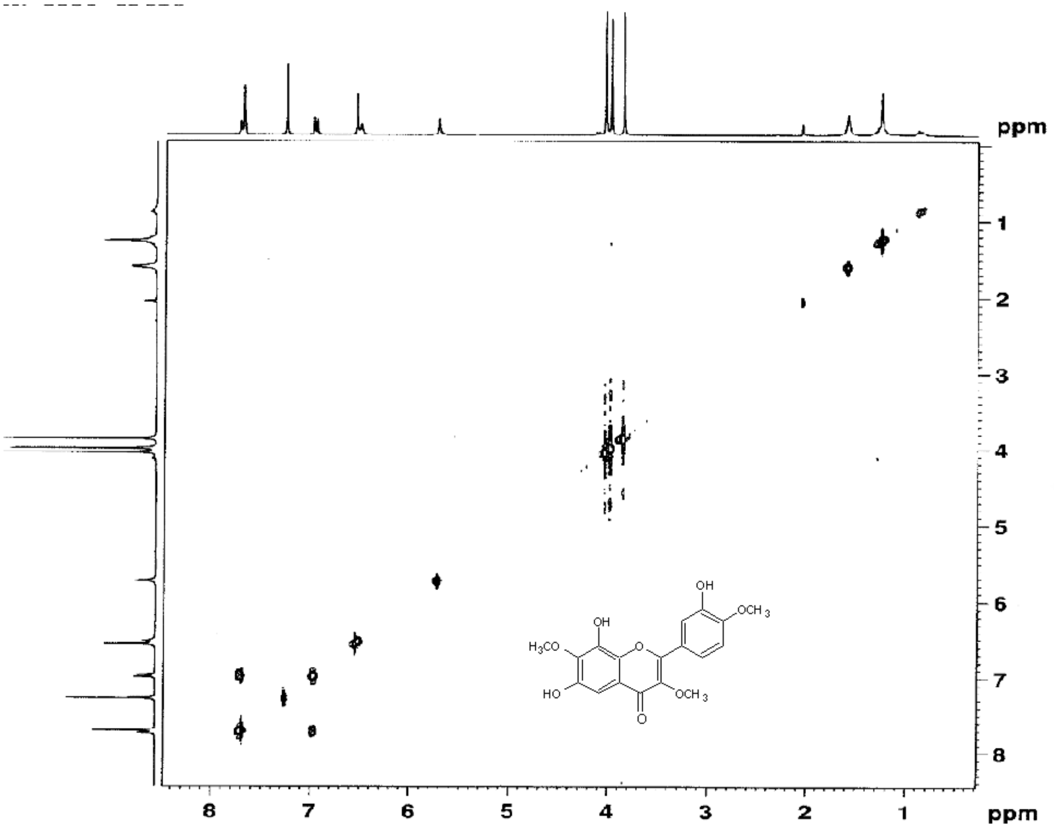

S12.HSQC spectrum of **Compound 2** in CD3OD

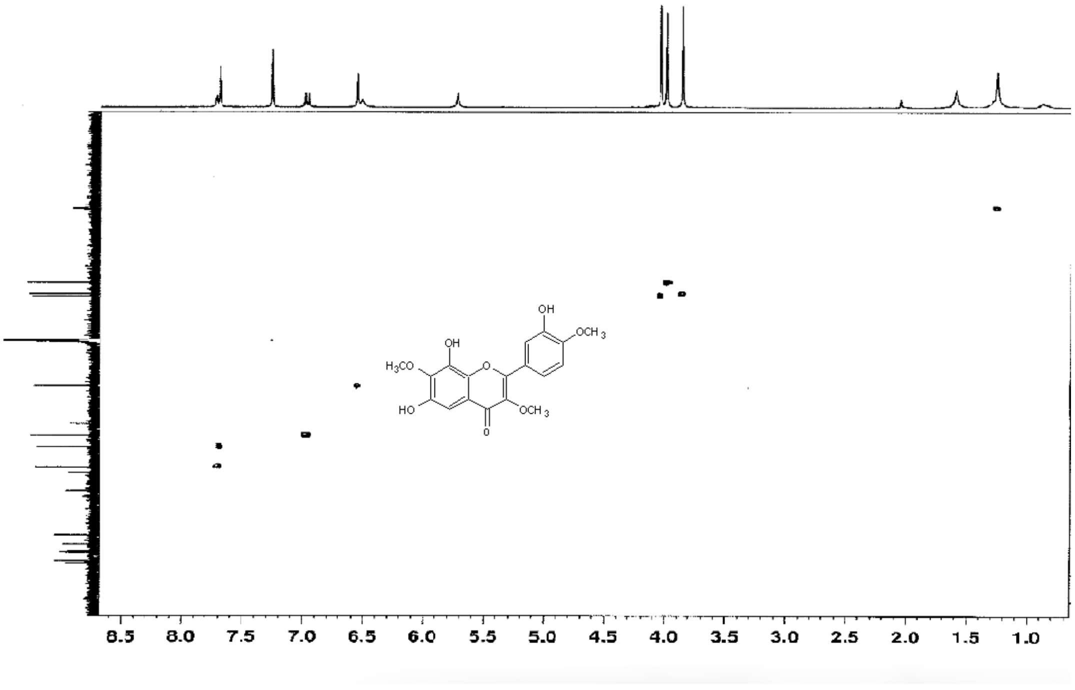

S13.HR-ESI-MS spectrum of **Compound 2**

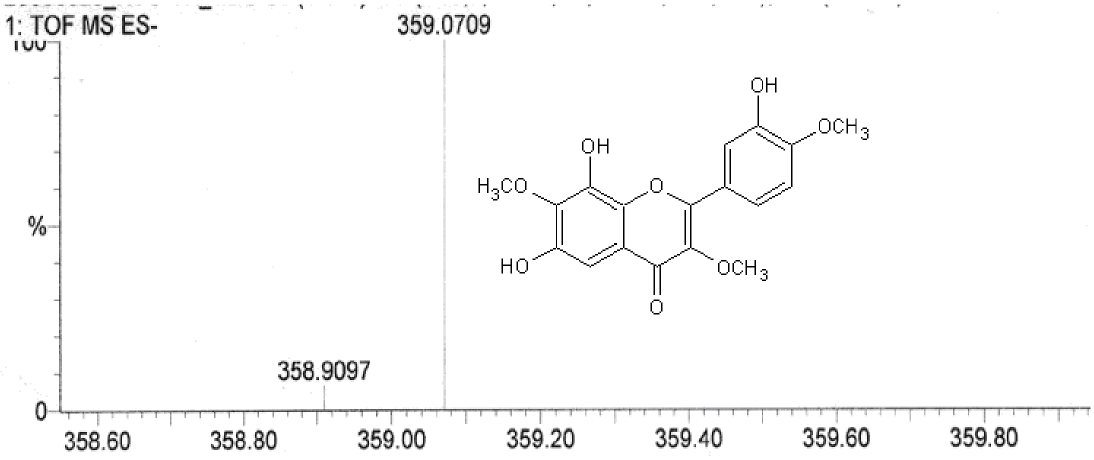

Supplement: Supplementary file 1 [file molecules-15-06357-s001.pdf]
